# The genome sequence of the whiskered bat, *Myotis mystacinus *(Kuhl, 1817)

**DOI:** 10.12688/wellcomeopenres.23345.1

**Published:** 2024-11-20

**Authors:** Hazel Ryan, Sonja C. Vernes, Emma C Teeling, Meike Mai, Natural History Museum Genome Acquisition Lab, Darwin Tree of Life Barcoding collective, Wellcome Sanger Institute Tree of Life Management, Samples and Laboratory team, Wellcome Sanger Institute Scientific Operations: Sequencing Operations, Wellcome Sanger Institute Tree of Life Core Informatics team, Tree of Life Core Informatics collective, Darwin Tree of Life Consortium

**Affiliations:** 1 Kent Bat Group, Whitstable, England, UK; 2 School of Biology, University of St Andrews, St Andrews, Scotland, UK; 3 Wellcome Sanger Institute, Hinxton, England, UK

**Keywords:** Myotis mystacinus, whiskered bat, genome sequence, chromosomal, Chiroptera

## Abstract

We present a genome assembly from an individual male *Myotis mystacinus* (whiskered bat; Chordata; Mammalia; Chiroptera; Vespertilionidae). The genome sequence has a total length of 2,081.20 megabases. Most of the assembly (97.52%) is scaffolded into 23 chromosomal pseudomolecules, including the X and Y sex chromosomes. The mitochondrial genome has also been assembled and is 16.93 kilobases in length.

## Species taxonomy

Eukaryota; Metazoa; Eumetazoa; Bilateria; Deuterostomia; Chordata; Craniata; Vertebrata; Gnathostomata; Teleostomi; Euteleostomi; Sarcopterygii; Dipnotetrapodomorpha; Tetrapoda; Amniota; Mammalia; Theria; Eutheria; Boreoeutheria; Laurasiatheria; Chiroptera; Yangochiroptera; Vespertilionoidea; Vespertilionidae; *Myotis* Kaup, 1829 (NCBI:txid98922)(subordinal taxonomy updated per Teeling *et al.*, 2005), *Myotis mystacinus* (Kuhl, 1817).

## Background

The whiskered bat, *Myotis mystacinus* (Kuhl, 1817), is a small bat, weighing 4–7 g, with a black face and ears, a long, pointed tragus and dark brown frizzy dorsal fur ( Dietz & Kiefer, 2016). It is one of three cryptic species which are described as the ‘small *Myotis*’ group in the UK ( Bat Conservation Trust, 2024). The slim, parallel-sided penis can be used to distinguish male whiskered from Brandt’s bat, *Myotis brandtii*, and the whiskered bat is larger than the alcathoe bat, *Myotis alcathoe*. Females can be differentiated using dentition. A fourth cryptic species, the Steppe Whiskered, *Myotis aurascens*, is found in Europe and can only be reliably separated from the other species by genetics.

The whiskered bat is listed as least concern on the IUCN Red list ( Coroiu, 2016), but local declines have been reported due to roost loss, disturbance in hibernation and conversion of orchards ( Dietz & Kiefer, 2016). It is a widespread species, found from Morocco to Southern Scandinavia and east to the Caucasus and Urals. The distribution and status of this species is likely to change with further genetic research and three subspecies have been described ( Wilson & Mittermeier, 2019).

This bat displays very manoeuvrable flight, foraging along hedges, forest edges and water bodies for flying insects such as small Diptera, Lepidoptera, Hymenoptera and lacewings. It is a crevice dweller, roosting in buildings under hanging tiles and roof slates with maternity colonies of 20 to 60 adult females ( Dietz & Kiefer, 2016). In winter it can be regularly found hibernating in caves, mines, tunnels and boulder heaps. It is one of the most abundant species found swarming at caves in autumn.

Sequencing of the genome is particularly important due to the confusion with similar cryptic species, uncertainty about whether the Balkan whiskered bat should be assigned to *Myotis mystacinus* or *Myotis aurascens*, and the possibility that there are further unrecognised *Myotis* species in this group. The haplotype-resolved genome presented here was sequenced as part of the Bat1K initiative ( Teeling *et al.*, 2018) and the Darwin Tree of Life project ( Darwin Tree of Life Project Consortium, 2022).

## Genome sequence report

**Figure 1.**
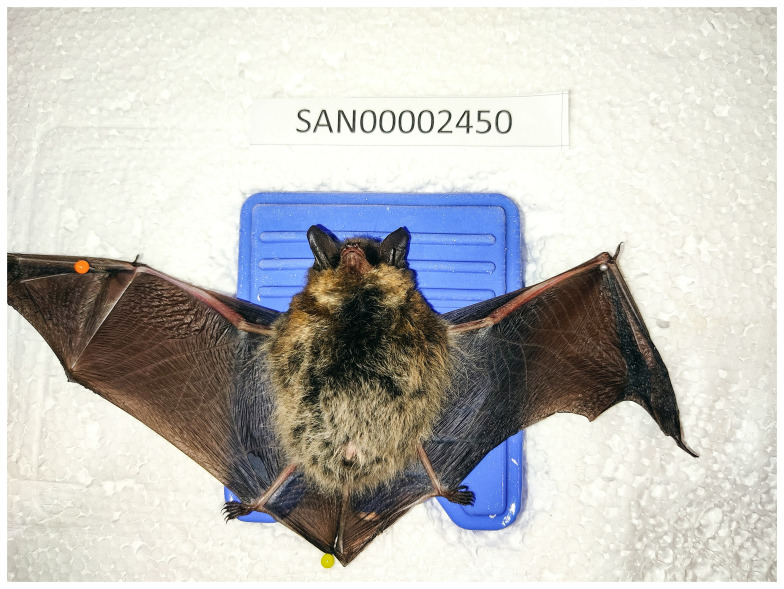
Photograph of the *Myotis mystacinus* (mMyoMys1) specimen used for genome sequencing.

The genome of *Myotis mystacinus* ( Figure 1) was sequenced using Pacific Biosciences single-molecule HiFi long reads, generating a total of 93.29 Gb (gigabases) from 9.44 million reads, providing an estimated 46-fold coverage. Primary assembly contigs were scaffolded with chromosome conformation Hi-C data, which produced 129.88 Gb from 860.14 million reads, yielding an approximate coverage of 62-fold. Specimen and sequencing details are summarised in Table 1.

**Table 1.** Specimen and sequencing data for *Myotis mystacinus*.

Project information			
**Study title**	*Myotis mystacinus* (whiskered bat)		
**Umbrella BioProject**	PRJEB73910		
**Species**	*Myotis mystacinus*		
**BioSample**	SAMEA114250307		
**NCBI taxonomy ID**	109479		
Specimen information			
**Technology**	**ToLID**	**BioSample** **accession**	**Organism part**
**PacBio long read sequencing**	mMyoMys1	SAMEA114250316	Other somatic animal tissue
**Hi-C sequencing**	mMyoMys1	SAMEA114250312	Heart
**RNA sequencing**	mMyoMys1	SAMEA114250316	Other somatic animal tissue
Sequencing information			
**Platform**	**Run accession**	**Read count**	**Base count (Gb)**
**Hi-C Illumina NovaSeq X**	ERR12765178	8.60e+08	129.88
**PacBio Revio**	ERR12760800	9.44e+06	93.29
**RNA Illumina NovaSeq X**	ERR13493928	1.12e+08	16.84

Assembly errors were corrected by manual curation, including 135 missing joins or mis-joins. This increased the assembly length by 1.05% and reduced the scaffold number by 3.13%. The final assembly has a total length of 2,081.20 Mb in 525 sequence scaffolds, with 1,566 gaps, and a scaffold N50 of 99.8 Mb ( Table 2).

**Table 2.** Genome assembly data for the *Myotis mystacinus* assembly.

Genome assembly	Haplotype 1	Haplotype 2
Assembly name	mMyoMys1.hap1.1	mMyoMys1.hap2.1
Assembly accession	GCA_964094495.2	GCA_964195625.1
Assembly level	chromosome	chromosome
Span (Mb)	2,081.2	1,943.3
Number of contigs	2,092	1,741
Number of scaffolds	525	327
Longest scaffold (Mb)	232.65	230.69
Assembly metrics *	Haplotype 1	Haplotype 2
Contig N50 length *(> 1 Mb)*	2.2 Mb	2.3 Mb
Scaffold N50 length *(= chromosome N50)*	99.8 Mb	98.8 Mb
Consensus quality (QV) ( *≥ 540)*	63.3	
*k*-mer completeness ( *≥ 95%)*	100.0%	
BUSCO ** (S > 90%, D < 5%)	C:95.8%[S:91.9%,D:3.9%], F:0.6%,M:3.6%,n:12,234	
Percentage of assembly mapped to chromosomes ( *≥ 90%)*	97.52%	
Sex chromosomes (localised homologous pairs)	X and Y	
Organelles (one complete allele)	Mitochondrial genome: 16.93 kb	

* Assembly metric benchmarks are adapted from Rhie *et al.* (2021) and the Earth BioGenome Project Report on Assembly Standards September 2024. ** BUSCO scores based on the laurasiatheria_odb10 BUSCO set using version 5.4.3. C = complete [S = single copy, D = duplicated], F = fragmented, M = missing, n = number of orthologues in comparison.

**Figure 2.**
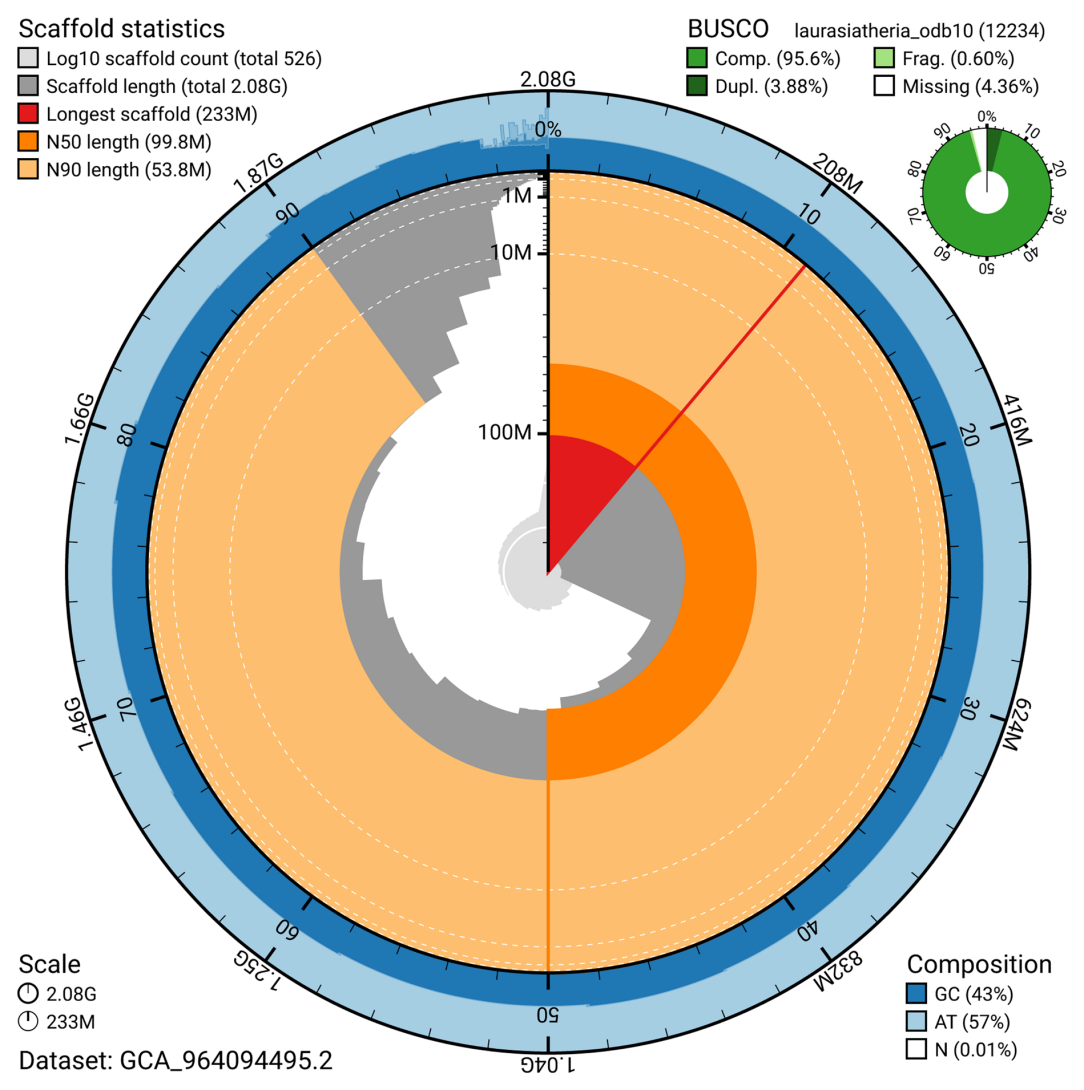
Genome assembly of *Myotis mystacinus*, mMyoMys1.hap1.1: metrics. The BlobToolKit snail plot provides an overview of assembly metrics and BUSCO gene completeness. The circumference represents the total length of the genome assembly, and the main plot is divided into 1,000 equal-sized bins around it. Scaffolds are arranged clockwise from largest to smallest and are depicted in dark grey. The longest scaffold is indicated by the red arc, while the deeper orange and pale orange arcs represent the N50 and N90 lengths, respectively. The light grey spiral at the centre illustrates the cumulative scaffold count on a logarithmic scale. The outermost blue tracks display the distribution of GC, AT, and N percentages across the bins. A summary of complete, fragmented, duplicated, and missing BUSCO genes in the laurasiatheria_odb10 set is presented at the top right. An interactive version of this figure is available at https://blobtoolkit.genomehubs.org/view/GCA_964094495.2/dataset/GCA_964094495.2/snail.

**Figure 3.**
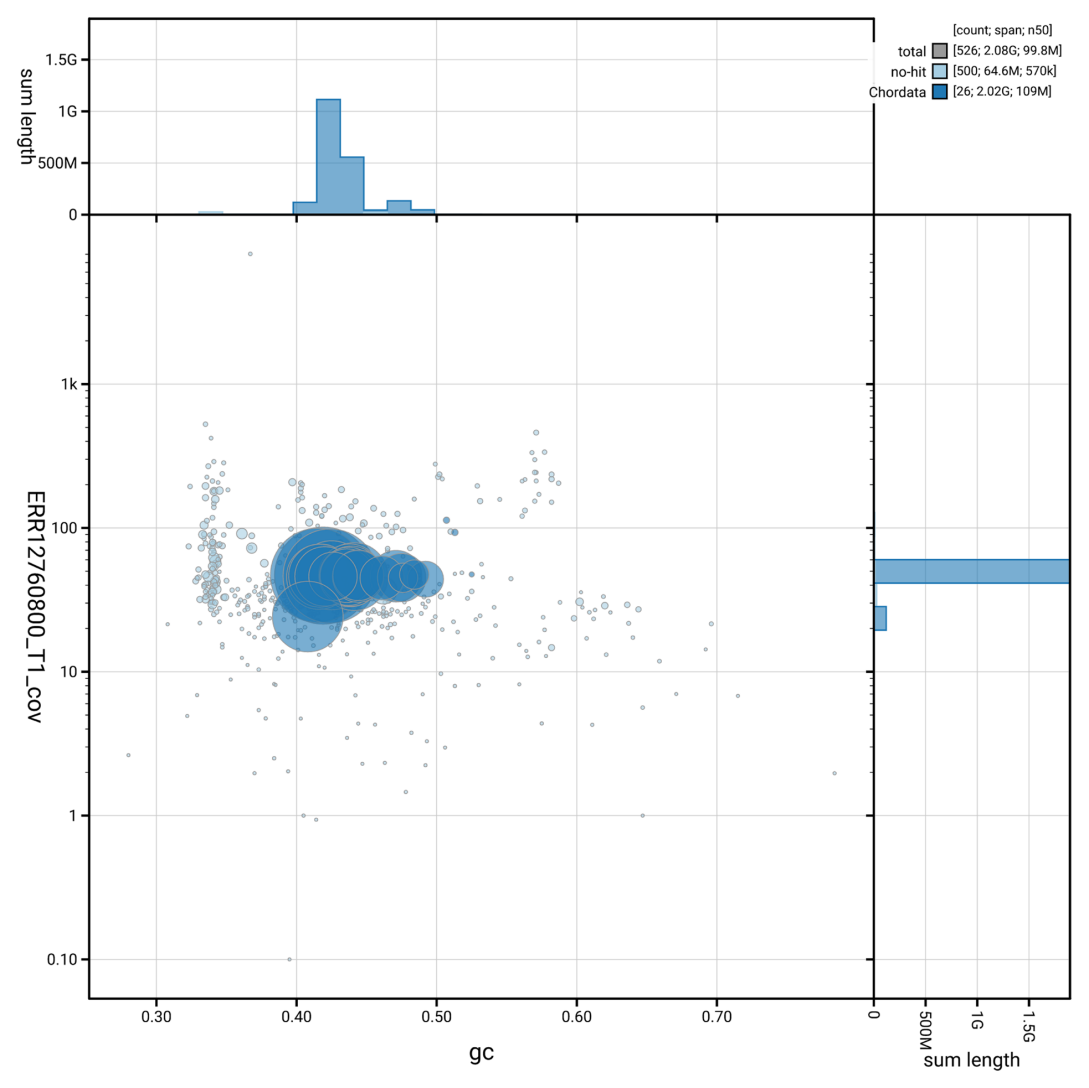
Genome assembly of *Myotis mystacinus*, mMyoMys1.hap1.1: BlobToolKit GC-coverage plot showing sequence coverage (vertical axis) and GC content (horizontal axis). The circles represent scaffolds, with the size proportional to scaffold length and the colour representing phylum membership. The histograms along the axes display the total length of sequences distributed across different levels of coverage and GC content. An interactive version of this figure is available at https://blobtoolkit.genomehubs.org/view/GCA_964094495.2/dataset/GCA_964094495.2/blob.

**Figure 4.**
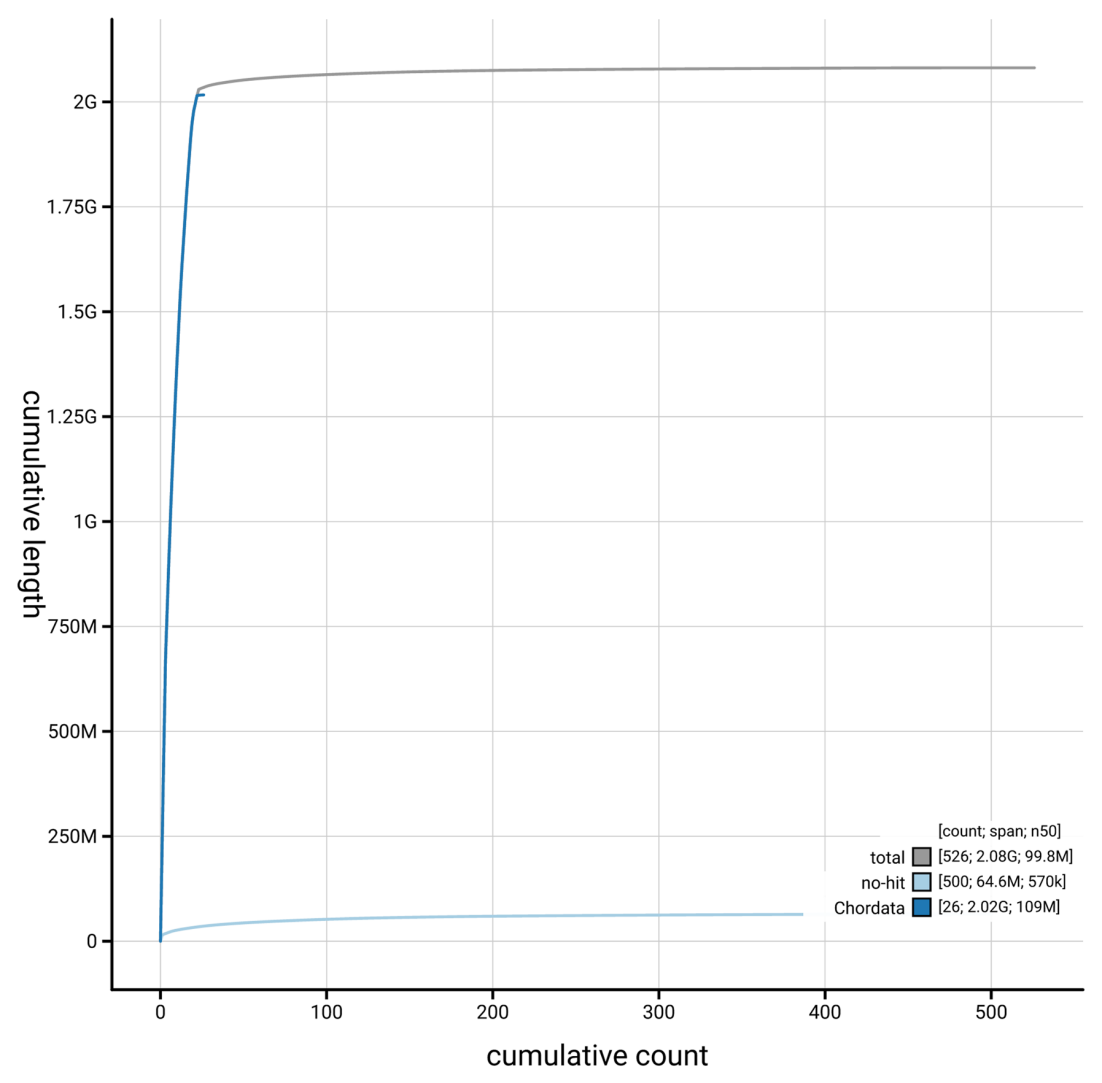
Genome assembly of *Myotis mystacinus* mMyoMys1.hap1.1: BlobToolKit cumulative sequence plot. The grey line shows cumulative length for all scaffolds. Coloured lines show cumulative lengths of scaffolds assigned to each phylum using the buscogenes taxrule. An interactive version of this figure is available at https://blobtoolkit.genomehubs.org/view/GCA_964094495.2/dataset/GCA_964094495.2/cumulative.

The snail plot in Figure 2 provides a summary of the assembly statistics, indicating the distribution of scaffold lengths and other assembly metrics. Figure 3 shows the distribution of scaffolds by GC proportion and coverage. Figure 4 presents a cumulative assembly plot, with separate curves representing different scaffold subsets assigned to various phyla, illustrating the completeness of the assembly.

**Figure 5.**
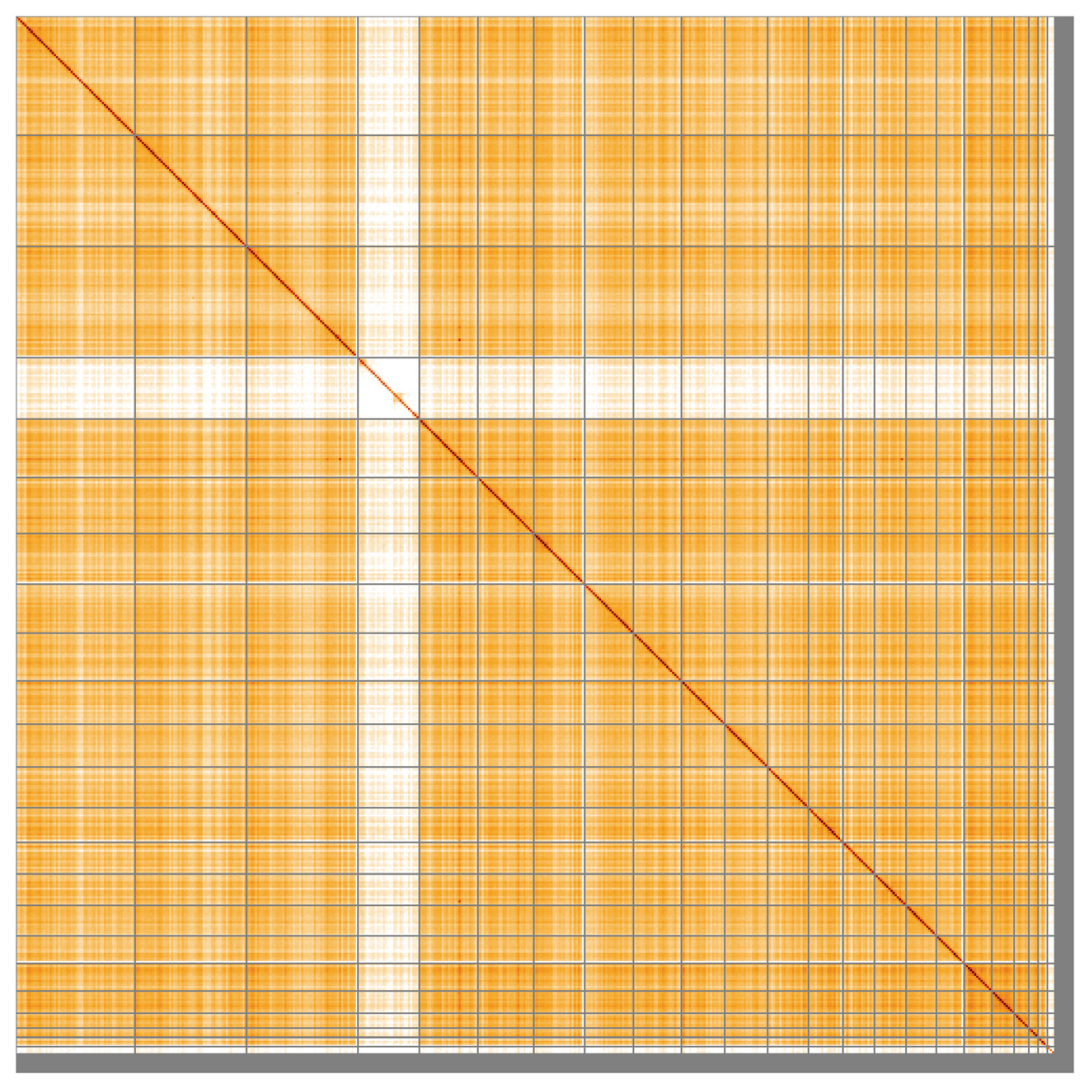
Genome assembly of *Myotis mystacinus* mMyoMys1.hap1.1: Hi-C contact map of the mMyoMys1.hap1.1 assembly, visualised using HiGlass. Chromosomes are shown in order of size from left to right and top to bottom. An interactive version of this figure may be viewed at https://genome-note-higlass.tol.sanger.ac.uk/l/?d=c77P6iz-Ryuja6G16EVKfg.

Most of the assembly sequence (97.52%) was assigned to 23 chromosomal-level scaffolds, representing 21 autosomes and the X and Y sex chromosomes. These chromosome-level scaffolds, confirmed by the Hi-C data, are named in order of size ( Figure 5; Table 3). Chromosomes X and Y were assigned based on read coverage and Hi-C data.

**Table 3.** Chromosomal pseudomolecules in the genome assembly of *Myotis mystacinus*, mMyoMys1.

Haplotype 1				Haplotype 2			
INSDC accession	Name	Length (Mb)	GC%	INSDC accession	Name	Length (Mb)	GC%
OZ065553.2	1	232.65	42.0	OZ077851.1	1	230.69	42.0
OZ065554.2	2	217.84	41.5	OZ077852.1	2	218.13	41.5
OZ065555.2	3	217.54	42.5	OZ077853.1	3	217.53	42.5
OZ065556.2	4	113.99	42.5	OZ077854.1	4	113.77	42.5
OZ065557.2	5	109.45	44.0	OZ077855.1	5	109.87	44.0
OZ065558.2	6	99.83	41.5	OZ077856.1	6	98.77	41.5
OZ065559.2	7	94.93	41.5	OZ077857.1	7	95.22	41.5
OZ065560.2	8	93.56	44.0	OZ077858.1	8	93.14	44.0
OZ065561.2	9	85.28	44.0	OZ077859.1	9	85.79	44.0
OZ065562.2	10	83.37	42.0	OZ077860.1	10	83.34	42.0
OZ065563.2	11	79.58	43.5	OZ077861.1	11	79.44	43.5
OZ065564.2	12	67.29	43.5	OZ077862.1	12	69.74	43.5
OZ065565.2	13	61.99	47.0	OZ077863.1	13	61.71	44.5
OZ065566.2	14	61.64	44.5	OZ077864.1	14	61.57	47.0
OZ065567.2	15	59.32	44.5	OZ077865.1	15	59.23	44.5
OZ065568.2	16	54.03	42.5	OZ077866.1	16	54.52	42.5
OZ065569.2	17	53.77	47.5	OZ077867.1	17	53.75	47.5
OZ065570.2	18	44.08	46.0	OZ077868.1	18	44.04	46.0
OZ065571.2	19	28.67	49.0	OZ077869.1	19	28.68	49.0
OZ065572.2	20	18.23	47.5	OZ077870.1	20	19.76	49.0
OZ065573.2	21	18.18	48.5	OZ077871.1	21	18.19	47.5
OZ075425.1	X	119.67	41.0				
OZ075426.1	Y	14.75	46.0				
OZ075427.1	MT	0.02	36.5				

While not fully phased, the assembly deposited is of one haplotype. Contigs corresponding to the second haplotype have also been deposited.

The mitochondrial genome was also assembled and can be found as a contig within the multifasta file of the genome submission, and as a separate fasta file with accession.

The final assembly has a Quality Value (QV) of 63.3 and *k*-mer completeness of 100.0%. BUSCO (v5.4.3) analysis using the laurasiatheria_odb10 reference set ( *n* = 12,234) indicated a completeness score of 95.8% (single = 91.9%, duplicated = 3.9%).

Metadata for specimens, BOLD barcode results, spectra estimates, sequencing runs, contaminants and pre-curation assembly statistics are given at https://links.tol.sanger.ac.uk/species/109479.

## Methods

### Sample acquisition and DNA barcoding

A male whiskered bat, *Myotis mystacinus*, (specimen ID NHMUK014446379, ToLID mMyoMys1) was collected from Halsford Green, East Grinstead, West Sussex on 19/10/22 by Helen Lucking, a member of Kent Bat Group. The specimen was found grounded with injuries consistent with capture by a domestic cat. It was passed to Hazel Ryan, a bat carer, on 21/10/22 as it required longer term care. It was prescribed a two-week course of antibiotics and anti-inflammatories by a vet due to an infection in the wrist and it was also given physiotherapy. After treatment, it was still unable to close the wing and so an X-ray was taken. This revealed a deep bone infection due to a fracture which could not be treated further and so the bat was euthanised on welfare grounds. Cervical dislocation was carried out on 19/11/22 followed by immediate dissection. The tissues were preserved by freezing. The specimen was cared for and identified by Hazel Ryan (Kent Bat Group). 

The initial identification was verified by an additional DNA barcoding process according to the framework developed by Twyford *et al.* (2024). A small sample was dissected from the specimens and stored in ethanol, while the remaining parts were shipped on dry ice to the Wellcome Sanger Institute (WSI). The tissue was lysed, the COI marker region was amplified by PCR, and amplicons were sequenced and compared to the BOLD database, confirming the species identification ( Crowley *et al.*, 2023). Following whole genome sequence generation, the relevant DNA barcode region was also used alongside the initial barcoding data for sample tracking at the WSI ( Twyford *et al.*, 2024). The standard operating procedures for Darwin Tree of Life barcoding have been deposited on protocols.io ( Beasley *et al.*, 2023).

### Nucleic acid extraction

The workflow for high molecular weight (HMW) DNA extraction at the Wellcome Sanger Institute (WSI) Tree of Life Core Laboratory includes a sequence of core procedures: sample preparation and homogenisation, DNA extraction, fragmentation and purification. Detailed protocols are available on protocols.io ( Denton *et al.*, 2023). The mMyoMys1 sample was prepared for DNA extraction by weighing and dissecting it on dry ice ( Jay *et al.*, 2023). Tissue was cryogenically disrupted using the Covaris cryoPREP ^®^ Automated Dry Pulverizer ( Narváez-Gómez *et al.*, 2023).

HMW DNA was extracted using the Automated MagAttract v2 protocol ( Oatley *et al.*, 2023a). DNA was sheared into an average fragment size of 12–20 kb in a Megaruptor 3 system ( Bates *et al.*, 2023). Sheared DNA was purified by solid-phase reversible immobilisation, using AMPure PB beads to eliminate shorter fragments and concentrate the DNA ( Oatley *et al.*, 2023b). The concentration of the sheared and purified DNA was assessed using a Nanodrop spectrophotometer and Qubit Fluorometer using the Qubit dsDNA High Sensitivity Assay kit. Fragment size distribution was evaluated by running the sample on the FemtoPulse system.

RNA was extracted from tissue of mMyoMys1 in the Tree of Life Laboratory at the WSI using the RNA Extraction: Automated MagMax™ *mir*Vana protocol ( do Amaral *et al.*, 2023). The RNA concentration was assessed using a Nanodrop spectrophotometer and a Qubit Fluorometer using the Qubit RNA Broad-Range Assay kit. Analysis of the integrity of the RNA was done using the Agilent RNA 6000 Pico Kit and Eukaryotic Total RNA assay.

### Hi-C preparation

Heart tissue from the mMyoMys1 sample was processed at the WSI Scientific Operations core, using the Arima-HiC v2 kit. Tissue (stored at –80 °C) was fixed, and the DNA crosslinked using a TC buffer with 22% formaldehyde. After crosslinking, the tissue was homogenised using the Diagnocine Power Masher-II and BioMasher-II tubes and pestles. Following the kit manufacturer's instructions, crosslinked DNA was digested using a restriction enzyme master mix. The 5’-overhangs were then filled in and labelled with biotinylated nucleotides and proximally ligated. An overnight incubation was carried out for enzymes to digest remaining proteins and for crosslinks to reverse. A clean up was performed with SPRIselect beads prior to library preparation.

### Library preparation and sequencing

Library preparation and sequencing were performed at the WSI Scientific Operations core. Pacific Biosciences SMRTbell libraries were constructed using the Revio HiFi prep kit, according to the manufacturers’ instructions. DNA sequencing was performed by the Scientific Operations core at the WSI on a Pacific Biosciences Revio instrument.

For Hi-C library preparation, DNA was fragmented to a size of 400 to 600 bp using a Covaris E220 sonicator. The DNA was then enriched, barcoded, and amplified using the NEBNext Ultra II DNA Library Prep Kit following manufacturers’ instructions. The Hi-C sequencing was performed using paired-end sequencing with a read length of 150 bp on an Illumina NovaSeq X instrument.

Poly(A) RNA-Seq libraries were constructed using the NEB Ultra II RNA Library Prep kit, following the manufacturer’s instructions. RNA sequencing was performed on the Illumina NovaSeq X instrument.

### Genome assembly, curation and evaluation

***Assembly***

The HiFi reads were assembled using Hifiasm ( Cheng *et al.*, 2021) in Hi-C phasing mode, where data were separated into two haplotypes. The Hi-C reads were aligned to the contigs using bwa-mem2 ( Vasimuddin *et al.*, 2019), and contigs were scaffolded with YaHS ( Zhou *et al.*, 2023), using the --break option for handling potential misassemblies. The resulting scaffolded assemblies were evaluated using Gfastats ( Formenti *et al.*, 2022), BUSCO ( Manni *et al.*, 2021) and MERQURY.FK ( Rhie *et al.*, 2020). The haplotypes were then combined for curation.

The mitochondrial genome was assembled using MitoHiFi ( Uliano-Silva *et al.*, 2023), which runs MitoFinder ( Allio *et al.*, 2020) and uses these annotations to select the final mitochondrial contig and to ensure the general quality of the sequence.

***Assembly curation***

The assembly was decontaminated using the Assembly Screen for Cobionts and Contaminants (ASCC) pipeline (article in preparation). Flat files and maps used in curation were generated in TreeVal ( Pointon *et al.*, 2023). Manual curation was primarily conducted using PretextView ( Harry, 2022), with additional insights provided by JBrowse2 ( Diesh *et al.*, 2023) and HiGlass ( Kerpedjiev *et al.*, 2018). Scaffolds were visually inspected and corrected as described by Howe *et al.* (2021). Any identified contamination, missed joins, and mis-joins were corrected, and duplicate sequences were tagged and removed. Sex chromosomes were identified by read coverage statistics. The curation process is documented at https://gitlab.com/wtsi-grit/rapid-curation (article in preparation).

***Evaluation of the final assembly***

The final assembly was post-processed and evaluated using the three Nextflow ( Di Tommaso *et al.*, 2017) DSL2 pipelines: sanger-tol/readmapping ( Surana *et al.*, 2023a), sanger-tol/genomenote ( Surana *et al.*, 2023b), and sanger-tol/blobtoolkit ( Muffato *et al.*, 2024). The readmapping pipeline aligns the Hi-C reads using bwa-mem2 ( Vasimuddin *et al.*, 2019) and combines the alignment files with SAMtools ( Danecek *et al.*, 2021). The genomenote pipeline converts the Hi-C alignments into a contact map using BEDTools ( Quinlan & Hall, 2010) and the Cooler tool suite ( Abdennur & Mirny, 2020). The contact map is visualised in HiGlass ( Kerpedjiev *et al.*, 2018). This pipeline also generates assembly statistics using the NCBI datasets report ( Sayers *et al.*, 2024), computes *k*-mer completeness and QV consensus quality values with FastK and MERQURY.FK, and runs BUSCO ( Manni *et al.*, 2021) to assess completeness.

The blobtoolkit pipeline is a Nextflow port of the previous Snakemake Blobtoolkit pipeline ( Challis *et al.*, 2020). It aligns the PacBio reads in SAMtools and minimap2 ( Li, 2018) and generates coverage tracks for regions of fixed size. In parallel, it queries the GoaT database ( Challis *et al.*, 2023) to identify all matching BUSCO lineages to run BUSCO ( Manni *et al.*, 2021). For the three domain-level BUSCO lineages, the pipeline aligns the BUSCO genes to the UniProt Reference Proteomes database ( Bateman *et al.*, 2023) with DIAMOND blastp ( Buchfink *et al.*, 2021). The genome is also divided into chunks according to the density of the BUSCO genes from the closest taxonomic lineage, and each chunk is aligned to the UniProt Reference Proteomes database using DIAMOND blastx. Genome sequences without a hit are chunked using seqtk and aligned to the NT database with blastn ( Altschul *et al.*, 1990). The blobtools suite combines all these outputs into a blobdir for visualisation.

The genome assembly and evaluation pipelines were developed using nf-core tooling ( Ewels *et al.*, 2020) and MultiQC ( Ewels *et al.*, 2016), relying on the Conda package manager, the Bioconda initiative ( Grüning *et al.*, 2018), the Biocontainers infrastructure ( da Veiga Leprevost *et al.*, 2017), as well as the Docker ( Merkel, 2014) and Singularity ( Kurtzer *et al.*, 2017) containerisation solutions.

Table 4 contains a list of relevant software tool versions and sources.

**Table 4.** Software tools: versions and sources.

Software tool	Version	Source
BEDTools	2.30.0	https://github.com/arq5x/bedtools2
BLAST	2.14.0	ftp://ftp.ncbi.nlm.nih.gov/blast/executables/ blast+/
BlobToolKit	4.3.7	https://github.com/blobtoolkit/blobtoolkit
BUSCO	5.4.3 and 5.5.0	https://gitlab.com/ezlab/busco
bwa-mem2	2.2.1	https://github.com/bwa-mem2/bwa-mem2
Cooler	0.8.11	https://github.com/open2c/cooler
DIAMOND	2.1.8	https://github.com/bbuchfink/diamond
fasta_windows	0.2.4	https://github.com/tolkit/fasta_windows
FastK	427104ea91c78c3b8b8b49f1a7d6bbeaa869ba1c	https://github.com/thegenemyers/FASTK
Gfastats	1.3.6	https://github.com/vgl-hub/gfastats
GoaT CLI	0.2.5	https://github.com/genomehubs/goat-cli
Hifiasm	0.19.8-r587	https://github.com/chhylp123/hifiasm
HiGlass	44086069ee7d4d3f6f3f0012569789ec138f42b84 aa44357826c0b6753eb28de	https://github.com/higlass/higlass
Merqury.FK	d00d98157618f4e8d1a9190026b19b471055b 22e	https://github.com/thegenemyers/MERQURY. FK
MitoHiFi	3	https://github.com/marcelauliano/MitoHiFi
MultiQC	1.14, 1.17, and 1.18	https://github.com/MultiQC/MultiQC
NCBI Datasets	15.12.0	https://github.com/ncbi/datasets
Nextflow	23.04.0-5857	https://github.com/nextflow-io/nextflow
PretextView	0.2.5	https://github.com/sanger-tol/PretextView
samtools	1.16.1, 1.17, and 1.18	https://github.com/samtools/samtools
sanger-tol/ ascc	-	https://github.com/sanger-tol/ascc
sanger-tol/ genomenote	1.1.1	https://github.com/sanger-tol/genomenote
sanger-tol/ readmapping	1.2.1	https://github.com/sanger-tol/readmapping
Seqtk	1.3	https://github.com/lh3/seqtk
Singularity	3.9.0	https://github.com/sylabs/singularity
TreeVal	1.0.0	https://github.com/sanger-tol/treeval
YaHS	1.2a.2	https://github.com/c-zhou/yahs

### Wellcome Sanger Institute – Legal and Governance

The materials that have contributed to this genome note have been supplied by a Darwin Tree of Life Partner. The submission of materials by a Darwin Tree of Life Partner is subject to the **‘Darwin Tree of Life Project Sampling Code of Practice’**, which can be found in full on the Darwin Tree of Life website here. By agreeing with and signing up to the Sampling Code of Practice, the Darwin Tree of Life Partner agrees they will meet the legal and ethical requirements and standards set out within this document in respect of all samples acquired for, and supplied to, the Darwin Tree of Life Project.

Further, the Wellcome Sanger Institute employs a process whereby due diligence is carried out proportionate to the nature of the materials themselves, and the circumstances under which they have been/are to be collected and provided for use. The purpose of this is to address and mitigate any potential legal and/or ethical implications of receipt and use of the materials as part of the research project, and to ensure that in doing so we align with best practice wherever possible. The overarching areas of consideration are:

•    Ethical review of provenance and sourcing of the material

•    Legality of collection, transfer and use (national and international)

Each transfer of samples is further undertaken according to a Research Collaboration Agreement or Material Transfer Agreement entered into by the Darwin Tree of Life Partner, Genome Research Limited (operating as the Wellcome Sanger Institute), and in some circumstances other Darwin Tree of Life collaborators.

## Data Availability

European Nucleotide Archive: Myotis mystacinus (whiskered bat). Accession number PRJEB73910; https://identifiers.org/ena.embl/PRJEB73910. The genome sequence is released openly for reuse. The *Myotis mystacinus* genome sequencing initiative is part of the Bat1K project and the Darwin Tree of Life (DToL) project. All raw sequence data and the assembly have been deposited in INSDC databases. The genome will be annotated using available RNA-Seq data and presented through the Ensembl pipeline at the European Bioinformatics Institute. Raw data and assembly accession identifiers are reported in Table 1 and Table 2.

## Author information

Members of the Natural History Museum Genome Acquisition Lab are listed here: https://doi.org/10.5281/zenodo.12159242.

Members of the Darwin Tree of Life Barcoding collective are listed here: https://doi.org/10.5281/zenodo.12158331.

Members of the Wellcome Sanger Institute Tree of Life Management, Samples and Laboratory team are listed here: https://doi.org/10.5281/zenodo.12162482.

Members of Wellcome Sanger Institute Scientific Operations: Sequencing Operations are listed here: https://doi.org/10.5281/zenodo.12165051.

Members of the Wellcome Sanger Institute Tree of Life Core Informatics team are listed here: https://doi.org/10.5281/zenodo.12160324.

Members of the Tree of Life Core Informatics collective are listed here: https://doi.org/10.5281/zenodo.12205391.

Members of the Darwin Tree of Life Consortium are listed here: https://doi.org/10.5281/zenodo.4783558.
