# Supplementary material for: The genome sequence of the whiskered bat, Myotis mystacinus (Kuhl, 1817)
Source: Wellcome Open Res. 2024 Nov 20;9:684. [Version 1] doi: 10.12688/wellcomeopenres.23345.1 (PMC11615438; doi:10.12688/wellcomeopenres.23345.1)
Supplement: Peer review history

# Reviewer report: Reviewer response for version 1

**DOI:** 10.21956/wellcomeopenres.25730.r112742

**Published:** 2024-12-03

**Authors:** Xiuguang Mao

**Affiliations:** 1 School of Ecological and Environmental Sciences, East China Normal University, Shanghai, China

Ryan et al. present a high-quality chromosome-scale assembly for *Myotis mystacinus *and this genomic resource, together with other published *Myotis* genomes, can be used to study the genome evolution and molecular adaptation in *Myotis*. For current version of manuscript, I only have two minor comments.

 1) In Table 2. “Consensus quality (QV) (≥ 540)” should be “Consensus quality (QV) (≥ 50)” ?

 2) In Methods: “Sex chromosomes were identified by read coverage statistics.” Could the authors provide more details for this?

Are sufficient details of methods and materials provided to allow replication by others?

Yes

Is the rationale for creating the dataset(s) clearly described?

Yes

Are the datasets clearly presented in a useable and accessible format?

Yes

Are the protocols appropriate and is the work technically sound?

Yes

Reviewer Expertise:

Comparative genomics, genome assembly, evolutionary biology, speciation, bats

I confirm that I have read this submission and believe that I have an appropriate level of expertise to confirm that it is of an acceptable scientific standard.

# Reviewer report: Reviewer response for version 1

**DOI:** 10.21956/wellcomeopenres.25730.r112744

**Published:** 2024-12-02

**Authors:** Jiang Feng

**Affiliations:** 1 Northeast Normal University, Changchun, China

This study reported a new genome assembly of the whiskered bat, *Myotis mystacinus* (Kuhl, 1817). The genome has not been previously published to this reviewer’s knowledge and may be an important resource for Chiroptera and future comparative studies. Especially, the whiskered bat, *Myotis mystacinus* (Kuhl, 1817) is one of three cryptic species which are described as the ‘small *Myotis*’ group in the UK, along with the Brandt’s bat, *Myotis brandtii* and the alcathoe bat, *Myotis alcathoe*. Their genomic genetic information is essential for studies on species identification, interspecies relationships, and evolutionary relationships. In general, this study would be of interest to those who are working on bats and biodiversity.  The methods are appropriate and the genome sequence report is concise.

**Some concerns and suggestions are listed as follows:**

 1.A number of studies have revealed that echolocation is central in shaping the diversification of bats. The whiskered bat, *Myotis mystacinus* (Kuhl, 1817) as you described in this study, who is one of three cryptic species along with *Myotis brandtii* and *Myotis alcathoe*. The echolocation call parameters of these three bat species should be introduced to provide a clearer understanding of their characteristics.

 2. The number of autosomes could be added in the Abstract, as “…23 chromosomal pseudomolecules, including 21 autosomes and the X and Y sex chromosomes.

 3.The collection and preservation conditions of various tissues used for DNA and RNA extraction experiments, etc., should be detailed in the Materials and Methods section. The information of tissue used for Hi-C sequencing and RNA sequencing should be described in Table 1.

Are sufficient details of methods and materials provided to allow replication by others?

Partly

Is the rationale for creating the dataset(s) clearly described?

Yes

Are the datasets clearly presented in a useable and accessible format?

Yes

Are the protocols appropriate and is the work technically sound?

Yes

Reviewer Expertise:

Animal ecology, Behavioral ecology, Population genetics

I confirm that I have read this submission and believe that I have an appropriate level of expertise to confirm that it is of an acceptable scientific standard.
